# Blocking Hepatoma-Derived Growth Factor Attenuates Vasospasm and Neuron Cell Apoptosis in Rats Subjected to Subarachnoid Hemorrhage

**DOI:** 10.1007/s12975-021-00928-y

**Published:** 2021-07-05

**Authors:** Chia-Li Chung, Chieh-Hsin Wu, Yu-Hua Huang, Shu-Chuan Wu, Chee-Yin Chai, Hung-Pei Tsai, Aij-Lie Kwan

**Affiliations:** 1 Graduate Institute of Medicine, College of Medicine, Kaohsiung Medical University, Kaohsiung, Taiwan; 2 Department of Surgery, Kaohsiung Municipal Siaogang Hospital, Kaohsiung, Taiwan; 3 Department of Neurosurgery, Kaohsiung Medical University Hospital, Kaohsiung, Taiwan; 4 Department of Surgery, College of Medicine, Kaohsiung Medical University, Kaohsiung, Taiwan; 5 Department of Neurosurgery, Kaohsiung Chang Gung Memorial Hospital and Chang Gung University College of Medicine, Kaohsiung, Taiwan; 6 Department of Pathology, Kaohsiung Medical University Hospital, Kaohsiung, Taiwan; 7 Department of Pathology, College of Medicine, Kaohsiung Medical University, Kaohsiung, Taiwan; 8 Division of Neurosurgery, Department of Surgery, Kaohsiung Medical University Hospital, Kaohsiung Medical University, No.100, Tzyou 1st Road, Kaohsiung, 80756 Taiwan; 9 Department of Neurosurgery, University of Virginia, Charlottesville, VA USA

## Abstract

Subarachnoid hemorrhage (SAH) is an important subcategory of stroke due to its unacceptably high mortality rate as well as the severe complications it causes, such as cerebral vasospasm, neurological deficits, and cardiopulmonary abnormality. Hepatoma-derived growth factor (HDGF) is a growth factor related to normal development and is involved in liver development and regeneration. This study explored the relationship between SAH and HDGF. Sixty rats were divided into five groups (n = 12/group): (A) control group; (B) rHDGF ab only group [normal animals treated with 50 µM recombinant HDGF antibodies (rHDGF ab)]; (C) SAH group; (D) SAH + pre-rHDGF ab group (SAH animals pre-treated with 50 µM rHDGF ab into the subarachnoid space within 24 h before SAH); and (E) SAH + post-rHDGF ab group (SAH animals post-treated with 50 µM rHDGF ab into the subarachnoid space within 24 h after SAH). At 48 h after SAH, serum and cerebrospinal fluid (CSF) samples were collected to measure the levels of pro-inflammatory factors by ELISA, and rat cortex tissues were used to measure protein levels by western blot analysis. Immunofluorescence staining for Iba-1, GFAP, TUNEL, and NeuN was detected proliferation of microglia and astrocyte and apoptosis of neuron cells. Neurological outcome was assessed by ambulation and placing/stepping reflex responses. Morphology assay showed that pre-treatment and post-treatment with rHDGF ab attenuated vasospasm after SAH. SAH up-regulated the levels of TNF-α, IL-1β, and IL-6 in both the CSF and serum samples, and both pre- and post-treatment with rHDGF ab inhibited the up-regulation of these pro-inflammatory factors, except for the serum IL-6 levels. Western blot analysis demonstrated that SAH up-regulated pro-BDNF and NFκB protein levels, and both pre- and post-treatment with rHDGF ab significantly reduced the up-regulation. The result from immunofluorescence staining showed that SAH induced proliferation of microglia and astrocyte and apoptosis of neuron cells. Both pre- and post-treatment with rHDGF ab significantly attenuated proliferation of microglia and astrocyte and inhibited apoptosis of neuron cells. Furthermore, treatment with rHDGF ab significantly improved neurological outcome. Blocking HDGF attenuates neuron cell apoptosis and vasospasm through inhibiting inflammation in brain tissue at early phase after SAH.

## Introduction

Early brain injury that occurs at the time of bleeding is the leading cause of mortality (30–70%) following subarachnoid hemorrhage (SAH) [1, 2]. SAH survivors are at risk of developing delayed cerebral vasospasm, delayed cerebral ischemia, or delayed ischemic neurological deficits during the hospital course [2]. Delayed vasospasm develops in approximately 70% of patients between 3 and 14 days after SAH [1, 2]. For decades, it has been considered as the single and the most important cause of delayed cerebral ischemia and poor outcome [3]. Even patients with favorable outcomes are frequently left with significant residual memory, reduced executive functioning, or language deficits [4]. Cerebral vasospasm following aneurysmal SAH is the leading cause of death and disability after aneurysm rupture [5]. Cerebral ischemia secondary to vasospasm occurs in 20 to 30% of these patients and has been correlated with a 1.5- to threefold increase in mortality in the first 2 weeks after SAH [3, 6]. Although cerebral vasospasm associated with SAH has been recognized for more than 50 years, adequate treatment is still illusive. Thence, the pathophysiological mechanism contributing to this form of arterial dysfunction is a topic of intense experimental study.

One of the major consequences of early brain injury is apoptosis, which is known to occur within minutes to 24 h after SAH [7]. Immune cells, including microglia and astrocytes, release pro-inflammatory factors to induce cell apoptosis and activation of transcription factor, resulting in positive feedbacks. Microglia and astrocytes also mediate neuropathic behavior by modulating the activity of spinal neurons to cause central sensitization, which has been associated with inflammatory neuropathies in autoimmune thyroid diseases, complex regional pain syndrome, osteoarthritis, rheumatoid arthritis, post-operative pain, and SAH [7, 8].

Hepatoma-derived growth factor (HDGF) is a 240 amino-acid protein isolated from human hepatoma cells [9]. Surface expressed nucleolin has recently been identified as an HDGF receptor [10]. During cell development, HDGF stimulates cell proliferation in fibroblasts, endothelial cells, and hepatoma cells [11]. It is also a growth factor related to tissue organogenesis and is involved in the development and regeneration of the liver [12–14], lungs [15, 16], kidney [17], heart [18], and the vascular system [19–21]. On the other hand, HDGF expression is associated with various malignant cancers, including hepatocellular carcinoma (HCC), gastric cancer, non-small cell lung cancer, pancreatic cancer, and melanoma [22, 23], to name a few. In addition, HDGF plays important roles in various cellular events, including ribosome biogenesis, RNA processing, DNA damage repair, and transcriptional regulation [17]. Furthermore, many studies indicate that HDGF is a mitogen with extracellular proliferative effects on hepatoma cells, fibroblasts, vascular smooth muscle cells, and endothelial cells [16]. However, no reports showed the relationship between HDGF and SAH. Hence, this was the focus of the present investigation.

## Materials and Methods

### Animal Preparation

The study procedures were executed in accordance with the protocol approved by the Committee of Institutional Animal Research at Kaohsiung Medical University. Male Sprague–Dawley rats were purchased from BioLasco (BioLasco Taiwan Co., Ltd., Taipei, Taiwan, authorized by Charles River Lab). After arriving at the Kaohsiung Medical University vivarium, the rats were acclimated for at least 1 week before being used in the experiment. All rats were housed at a constant temperature (24 °C) and at regular light/dark cycles between 6:00 am and 6:00 pm, with free access to a standard diet. Animals weighing between 350 and 450 g were used in this study.

### SAH Induction

The one-shot SAH model was utilized. Briefly, rats were anesthetized by intraperitoneal injection of 40 mg/kg Zoletil 50® containing a mixture of zolazepam and tiletamine hypochloride (Virbac, Carros, France). The head was fixed in a stereotactic apparatus (Stoelting, Wood Dale IL, USA), and a 25-gauge butterfly needle was advanced into the cisterna magna to withdraw 0.3 mL of cerebrospinal fluid (CSF). Fresh, autologous, and non-heparinized blood (0.1 mL/100 g of body weight) drawn from the central tail artery was then slowly instilled into the subarachnoid space through a butterfly needle and tubing. Afterwards, the animals were kept in a ventral recumbent position for at least 30 min to promote ventral blood distribution. The morbidity caused by SAH was 100%, and the mortality was 0% in this study. The respiratory pattern of rats was inspected closely, and mechanical ventilation was provided if necessary. Upon fully awakening, the animals were sent back to the vivarium. In this study, there are five groups: (1) control group; (2) rHDGF ab group, treatment with rHDGF antibody into the subarachnoid space; (3) SAH group; (4) SAH + pre-rHDGF ab group, treatment with rHDGF antibody into the subarachnoid space within 24 h before SAH; and (5) SAH + post-rHDGF ab group, treatment with rHDGF antibody into the subarachnoid space within 24 h after SAH. All rats were used following randomization.

### Preparation of HDGF Hyperimmune Serum (Recombinant HDGF Antibody)

HDGF hyperimmune serum was customized by Leadgene Biomedical Inc. (Tainan, Taiwan). Briefly, 6- to 8-week-old BALB/c mice were primed by intraperitoneal injection of 50 μg of recombinant rat HDGF (rHDGF) protein in complete Freund’s adjuvant. Two weeks after the first injection, the mice were given another injection of 25 μg rHDGF antibody in PBS. The procedure was repeated at weeks 4 and 5 after antigen priming. The sera were collected and stored at – 20 °C until use.

### Neurological Evaluation

We followed the method of neurobehavioral evaluation from Dr. Huang et al. (2017) [24]. Neurobehavioral evaluation of animals was performed by assessing the sensorimotor integration of the forelimb and hind-limb activities using a modified limb-placing test that consisted of ambulation as well as placing and stepping reflex [25]. Motor deficit index (MDI) represented the sum of scores for walking by lower limbs and for placing/stepping response and was determined at 48 h after induction of SAH. Higher MDI values indicated poorer neurological outcomes. In addition, neurological evaluation was used as a double-blind trial.

### Tissue Processing

This protocol was conducted in a as previously described in Dr. Huang et al. (2017) [24]. At the end of experiments, each animal was anesthetized again for perfusion and fixation. The thoracic cage was opened, and the left ventricle was canalled using a No. 16 catheter. After clamping, the descending aorta and puncturing the right atrium, the brain was perfused with 180 mL of 2% paraformaldehyde and then 100 mL of phosphate buffer (0.01 M) under 36 °C and 100 mm Hg perfusion pressure. Gross inspection of harvested brains was performed to confirm the presence of subarachnoid blood clots over the basilar artery (BA), and the specimen was immersed in a fixative solution. The BAs were then separated from the brainstems, and the middle third of each vessel was dissected out. These arterial segments were flat-embedded in paraffin, and BA cross sections were cut into 3-μm sections and were stained with hematoxylin and eosin stain for subsequent analysis.

### Morphometric Assessment of BA

This protocol was conducted in a as previously described in Dr. Huang et al. (2017) [24]. Three cross sections from the middle-third BA in each animal were analyzed by a trained research staff blinded to the experimental groups. The thickness of BA was defined as the largest vertical distance between the inner surface of endothelium and the outer surface of adventitia. The arterial cross-sectional area was calculated using computer-based morphometric analysis (Image J; Universal Imaging Corp., USA). The average area of BA cross sections from each rat was calculated to obtain mean values for the degree of vasospasm at 48 h after SAH.

### Immunofluorescence Staining

After deparaffinization and rehydration, paraffin-embedded brain samples were heated by steam for 30 min to retrieve antigen using DAKO antigen retrieval solution (DAKO, Carpenteria, CA). Slides were then washed twice with Tris-buffered saline (TBS) and immersed in a 3% hydrogen peroxide solution for 10 min to inhibit endogenous peroxidase. Upon washing twice in TBS, the sections were incubated with TUNEL kit, mouse anti-NeuN (Merck; MAB377; Germany), mouse anti-GFAP (Sigma; G3893; USA), and rabbit anti-Iba1 (proteintech; 10,904–1-AP; USA) antibodies at room temperature to detect the presence of the cell number of DNA damage, astrocytes, microglia, and neuron cells, respectively. Slides were again washed twice with TBS and subsequently incubated with anti-rabbit antibody (Thermo; A11008; USA) and anti-mouse antibody (Thermo; A10524; USA) for 3 h at room temperature. Afterwards, the slides were washed twice with TBS, stained and mounted within Fluroshield™ with DAPI (Sigma; F6057; USA), and take a picture following LSM 700 confocal microscope.

### Western Blot

Rat cortex samples were collected at 48 h after SAH. Tissue extracts were prepared in 1 mL of ice-cold lysis buffer [50 mM Tris–HCl, pH 7.4, 0.25 M NaCl, 0.1% Nonidet P-40, 5 mM EDTA, 50 mM NaF, 1 × cocktail of protease inhibitors (Sigma, St. Louis, MO), 1 mM phenylmethylsulfonyl fluoride, and 1 mg/L aprotinin] and incubated on ice for 30 min. After centrifugation at 8000 g for 20 min at 4 ℃, protein amount in the supernatant was quantified using a protein assay kit from Bio-Rad Laboratories, and 60 μg of protein samples were separated by 12% SDS–polyacrylamide gel electrophoresis. Proteins were then transferred to nitrocellulose membranes. The membranes were blocked in PBS containing 5% fat-free milk for 90 min at room temperature and then incubated with antibodies (HDGF and cleaved caspase-3) for 2 h at room temperature. After washing, the membranes were incubated for 1 h at 25 °C with the appropriate horseradish peroxidase-labeled secondary antibodies, and the bound antibodies were visualized and quantified by chemiluminescence detection. The expression level of proteins of interest was normalized to the densitometric units of β-actin.

### ELISA

Serum and cerebrospinal fluid (CSF) samples were collected at 48 h after SAH, centrifuged immediately at 2000 × *g* for 10 min at 4 °C to remove cells, and were stored below − 15 °C prior to analysis. X These samples were concentrated by passing through C2 columns (Amersham, Nutley, USA), and the levels of TNF-α, IL-1β, and IL-6 were determined using an inflammatory factor ELISA system (Amersham) at 450 nm.

### Statistics

The results were analyzed using SPSS version 20.0 (IBM SPSS Statistics, location). Data were presented as mean ± standard deviation (SD). Group results were compared using Student’s *t* test, Mann–Whitney *U* test, or one-way analysis of variance (ANOVA). A *P* value of < 0.05 was considered statistically significant.

## Results

### Neurological Outcomes

The neurobehavioral scores, including ambulation, placing/stepping reflex, and MDI, were not different between the control and rHDGF ab only groups (Table 1). In animals subjected to SAH, both the ambulation (1.75 ± 0.46) and placing/stepping reflex (1.88 ± 0.35) scores were significantly higher than the non-SAH animals. Pre-treatment with rHDGF ab significantly decreased both the ambulation (1.12 ± 0.64; P < 0.05) and the placing/stepping reflex (1.25 ± 0.71) scores when compared with the SAH group. In addition, post-treatment with rHDGF ab significantly also decreased both the ambulation (1.38 ± 0.52; P < 0.05) and the placing/stepping reflex (1.25 ± 0.46) scores when compared with the SAH group. Likewise, MDI in the rHDGF ab pre-treatment group (2.38 ± 1.19; P < 0.05) and post-treatment group (2.63 ± 0.74; P < 0.05) were also significantly reduced when compared with the SAH group (3.63 ± 0.74) (Table 1).

**Table 1:** Behavioral assessment

Treatment	Ambulation	Placing/stepping reflex	MDI
Control	0	0	0
rHDGF ab	0	0	0
SAH	1.75 ± 0.46	1.88 ± 0.35	3.63 ± 0.74
SAH + pre-rHDGF ab	1.12 ± 0.64*	1.25 ± 0.71*	2.38 ± 1.19*
SAH + post-rHDGF ab	1.38 ± 0.52*	1.25 ± 0.46*	2.63 ± 0.74*

Neurological outcome was assessed by ambulation and placing/stepping reflex responses within 48 h after inducing SAH. MDI, motor deficit index, which is the sum of ambulation (walking with lower extremities) and placing/stepping reflex (dragging the dorsum of hind paw over the edge of a surface) scores

### Morphological Changes in BA

**Fig. 1:**
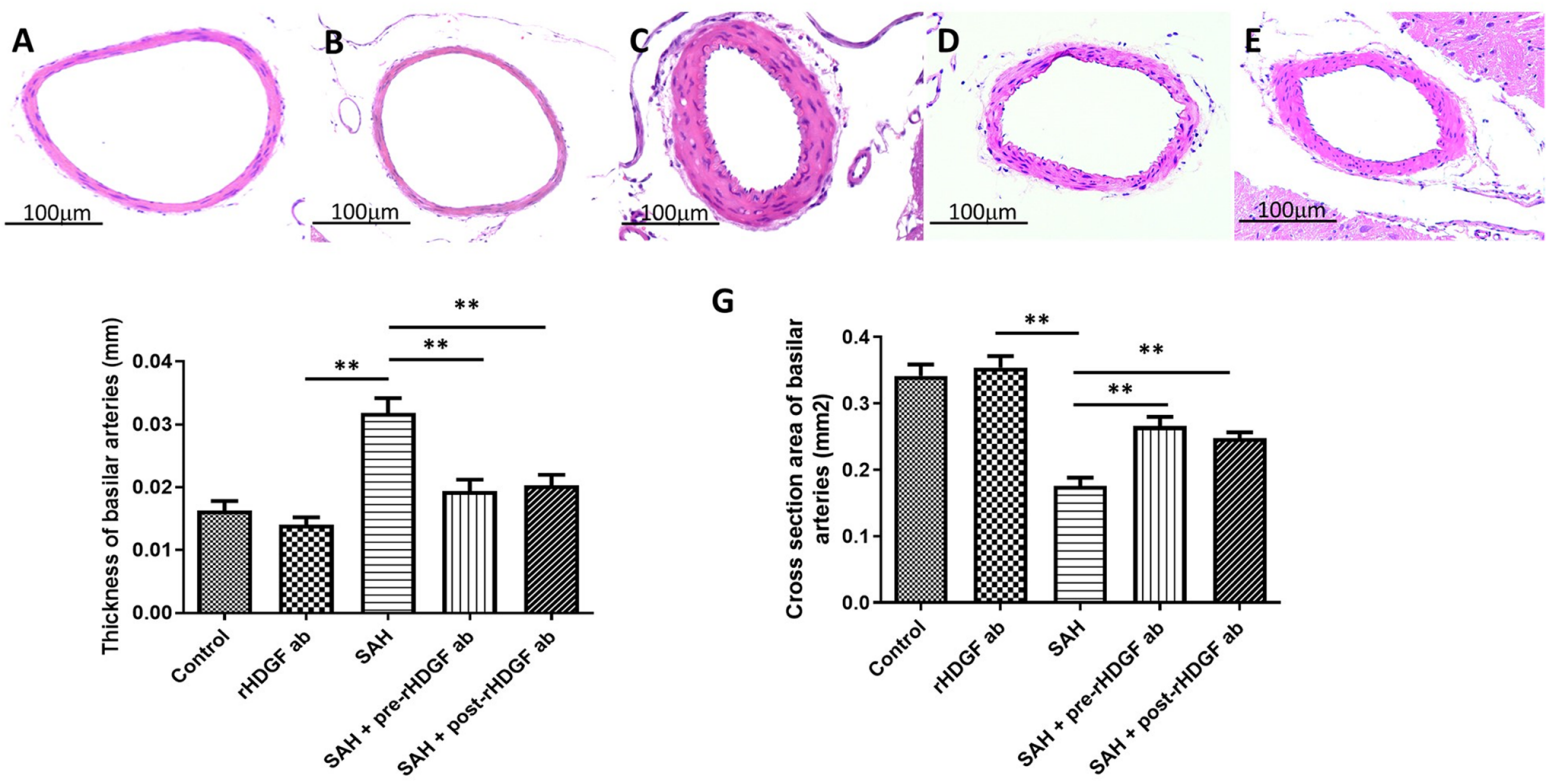
Representative micrographs of BA cross sections obtained from (**A**) control group, (**B**) rHDGF ab only group, (**C**) SAH group, (**D**) SAH + pre-rHDGF ab group, and (**E**) SAH + post-rHDGF ab group. (**F**) Comparison of the BA cross-sectional area among control, rHDGF ab, SAH, SAH + pre-rHDGF ab, and SAH + post-rHDGF ab group. (**G**) Comparison of the thickness of BA among the same four groups. All values are mean ± SD (n = 6). **P < 0.01

Upon microscopic examination, endothelial deformation, twisting of internal elastic laminae, and smooth muscle necrosis were seen in the BAs of rats subjected to SAH (compare Fig. 1C, D and E with Fig. 1A), while the morphology of animals in the rHDGF ab only group (Fig. 1B) was not visually different from that of the normal control (Fig. 1A).

### Cross-Sectional Area Changes in BA

The mean cross-sectional area of BA was 0.34 ± 0.045 mm^2^ in the control group and was 0.35 ± 0.044 mm^2^ in the rHDGF ab only group (Fig. 1E); there was no significant difference between these two groups. In animals with SAH, the BA cross-sectional area (0.177 ± 0.033 mm^2^) was reduced by 51.8% (P < 0.01) and 50.3% (P < 0.01) when compared with that in the normal control and rHDGF ab only groups, respectively. Both pre-treatment with rHDGF ab (0.266 ± 0.038 mm^2^; P < 0.05) and post-treatment with rHDGF ab (0.248 ± 0.022 mm^2^; P < 0.05) significantly increased the BA cross-sectional area when compared with that of the SAH only group (Fig. 1E).

### Changes in the Thickness of BA

There was no significant difference in the thickness of BA between the normal control (0.0163 ± 0.004 mm) and rHDGF ab only (0.0140 ± 0.003 mm) groups (Fig. 1F). A significant increase in the thickness of BA was found in the SAH group (0.0319 ± 0.006 mm; P < 0.01 vs both control and rHDGF ab groups). In SAH rats pre-treated with rHDGF ab, the thickness of BA was significantly reduced (0.0194 ± 0.005 mm; P < 0.01) when compared with SAH rats without treatment (Fig. 1F). In addition, In SAH rats post-treated with rHDGF ab, the thickness of BA was also significantly reduced (0.0202 ± 0.004 mm; P < 0.01) when compared with SAH rats without treatment (Fig. 1F).

### Proliferation of Microglia and Astrocytes

**Fig. 2:**
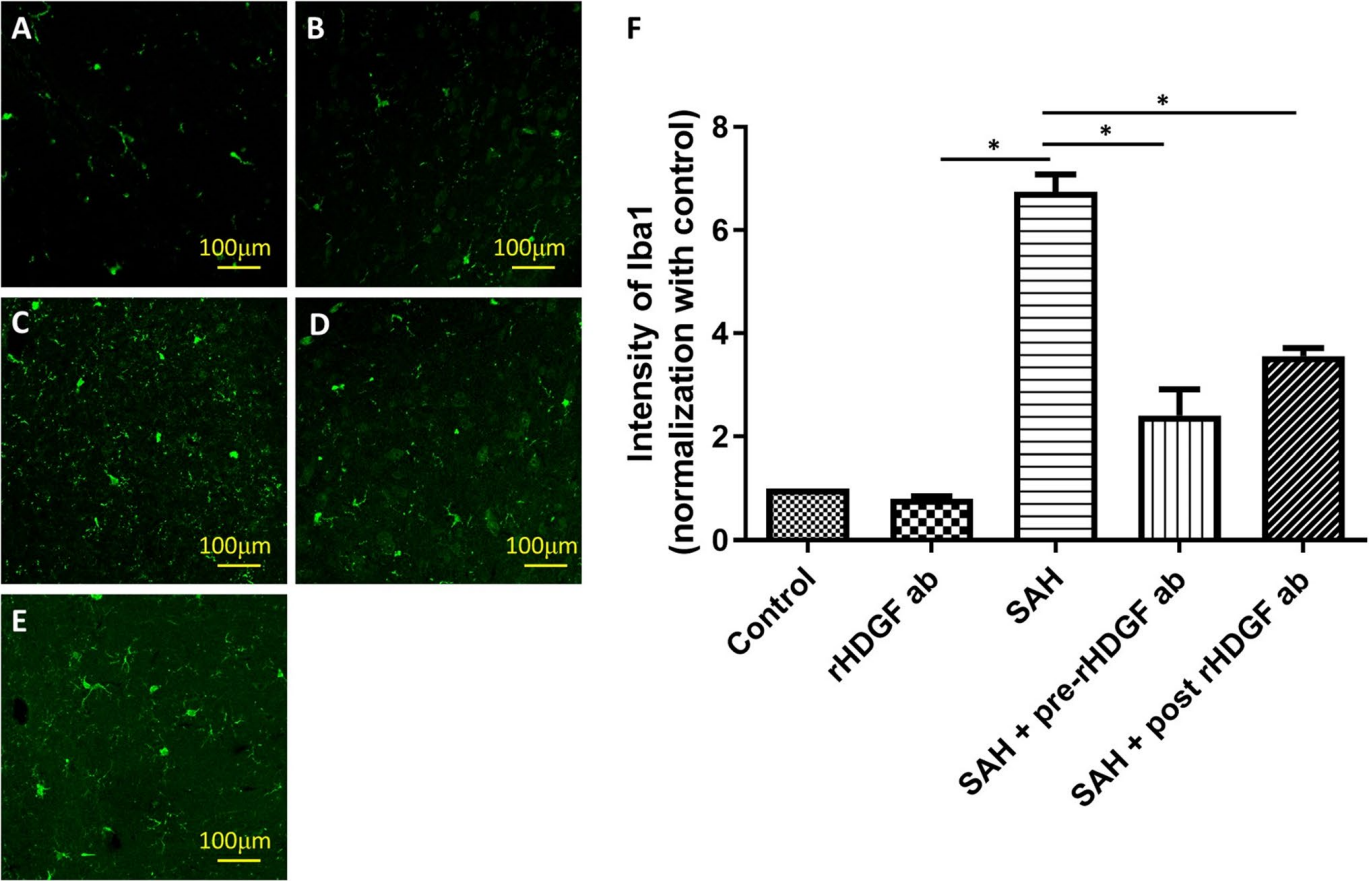
Proliferation of microglia in the rat brain as determined by immunofluorescence staining for Iba-1. Representative micrographs of Iba-1 staining are shown for: (**A**) control group, (**B**) rHDGF ab only group, (**C**) SAH group, (**D**) SAH + pre-rHDGF ab group, and (**E**) SAH + post-rHDGF ab group. (**F**) The intensities of immunofluorescence staining in the images were quantified relative to the levels of the control animals. All values are mean ± SD (n = 12). *P < 0.05

Besides in the bleeding area, activated microglia were found to diffuse into the brain parenchymal such as brain stem, cortex, and hippocampus [26, 27]. Likewise, after SAH attack, astrocytes were shown to be activated as a part of gliosis [28]. In this study, we used immunofluorescence staining for Iba-1 and GFAP to detect the presence of microglia cells and astrocytes, respectively. Immunofluorescence staining for Iba-1 was not significantly different in the control and the rHDGF ab only groups (compare Fig. 2A and B). SAH induced proliferation of microglia cells in the rat brain (Fig. 2C, D, and E). Quantitative analysis of the intensity of Iba-1 staining also revealed comparable levels between the control (set at 1.0) and the rHDGF ab only (1.10 ± 0.22) groups. By contrast, Iba-1 staining in the brain of the SAH rats was substantially elevated (8.53 ± 1.01), and both pre-treatment with rHDGF ab (4.83 ± 1.40, P < 0.05 compared with the SAH only group) and post-treatment with rHDGF ab (5.32 ± 0.78, P < 0.05 compared with the SAH only group) significantly reduced the proliferation of microglia cells. (Fig. 2E).

**Fig. 3:**
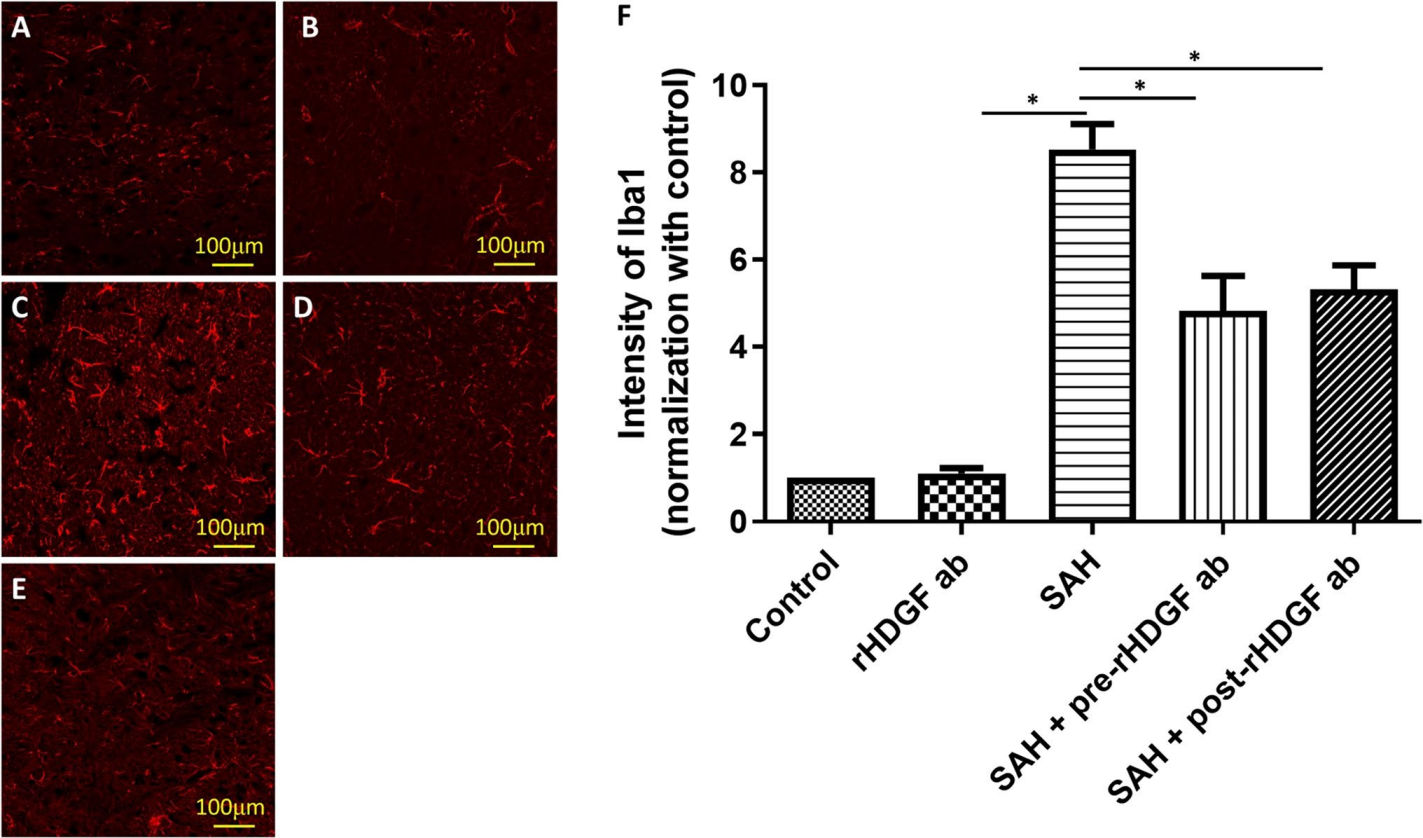
Proliferation of astrocytes in the rat brain as determined by immunofluorescence staining for GFAP. Representative micrographs of GFAP staining are shown for: (**A**) control group, (**B**) rHDGF ab only group, (**C**) SAH group, (**D**) SAH + pre-rHDGF ab group, and (**E**) SAH + post-rHDGF ab group. (**F**) The intensities of immunofluorescence staining in the images were quantified relative to the levels of the control animals. All values are mean ± SD (n = 12). *P < 0.05

The results of immunofluorescence staining for GFAP mirrored those of the Iba-1 staining; i.e., no significant difference in GFAP staining was found between the control and the rHDGF ab only groups (compare Fig. 3A and B), whereas SAH induced proliferation of astrocytes in the rat brain (Fig. 3C, D, and E). The intensities of GFAP staining the rHDGF ab only, SAH, SAH + pre-rHDGF ab, and SAH + post-rHDGF ab groups were 0.81 ± 0.08, 6.74 ± 0.59, 2.41 ± 0.88, and 3.56 ± 0.22, respectively, relative to the value of 1.0 set for the control group. No significant difference was seen between the control and rHDGF ab only groups. The intensity of GFAP staining was significantly elevated in the SAH group (P < 0.05, compared with the control), and this value was significantly reduced in SAH rats pre-treated with rHDGF ab (P < 0.05) and in SAH rats post-treated with rHDGF ab (P < 0.05) (Fig. 3E).

### ELISA of Pro-inflammatory Factors

**Fig. 4:**
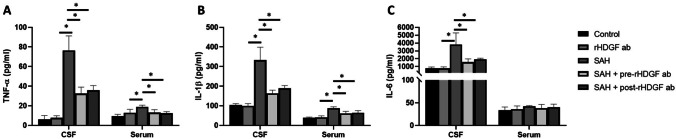
ELISA assay for pro-inflammatory factors in the CSF and Serum of rats following SAH. The samples were collected at 48 h after SAH. The levels of (**A**) TNF-α, (**B**) IL-1β, and (**C**) IL-6 were measured using commercially available kits. All values are mean ± SD (n = 12). *P < 0.05

To investigate the relationship between pro-inflammatory factors and SAH, the levels of TNF-α, IL-1β, and IL-6 in the CSF and serum samples were examined using ELISA at 6 h after SAH. No significant differences in any of the pro-inflammatory factor protein levels between the control and the rHDGF ab only groups were found in the CSF or serum samples (Fig. 4). In this early phase of SAH, expressions of TNF-α, IL-1β, and IL-6 in SAH group were significantly higher than those of the rHDGF ab only group in CSF (Fig. 4). In addition, expressions of TNF-α and IL-1β in SAH group were likewise significantly higher than those of the rHDGF ab only group in serum, whereas no significant difference in the IL-6 was seen in these samples. All of the aforementioned increases in protein expression were significantly attenuated (P < 0.05, compared with the respective SAH group upon pre-treatment with rHDGF ab and post-treatment with rHDGF ab) (Fig. 4).

### Western Blot Analysis

**Fig. 5:**
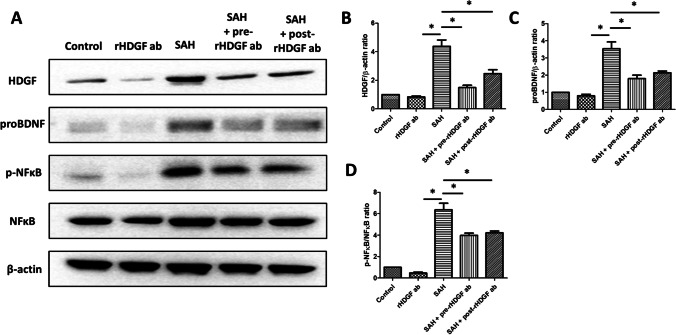
Western blot analysis to show the effect of rHDGF ab pre-treatment on the levels of HDGF, proBDNF, p-NFκB, and NFκB protein in the cortex of rats at 48 h following SAH. (**A**) Representative results of Western blots. The HDGF (**B**), proBDNF (**C**), and p-NFκB/NFκB (**D**) expression levels were normalized using internal control (β-actin). All values are mean ± SD (n = 12)

Brain-derived neurotrophic factor (BDNF) is a molecule that regulates neuronal survival and differentiation. BDNF is initially synthesized as a precursor, proBDNF, which is trafficked to the regulated secretory pathway [29]. NF-κB is a nuclear transcription factor that acts as a key regulator of both inflammatory response and cell death [30, 31]. Protein expressions of HDGF, proBDNF, p-NFκB (phosphorylated NFκB), and NFκB in the cortex were assessed at 48 h after SAH. Similar to the results obtained in other measurements, no significant differences in the expression levels of these proteins between the control and the rHDGF ab only groups were found, while they were all significantly increased in the SAH group when compared with their respective controls (Fig. 5). Pre-treatment with rHDGF antibody resulted in significant decreases in the levels of proBDNF, p-NFκB/NFκB, and HDGF when compared with the respective SAH groups (Fig. 5). In addition, post-treatment with rHDGF antibody also resulted in significant decreases in the levels of proBDNF, p-NFκB/NFκB, and HDGF when compared with the respective SAH groups.

### DNA Damage of Neuron Cells

**Fig. 6:**
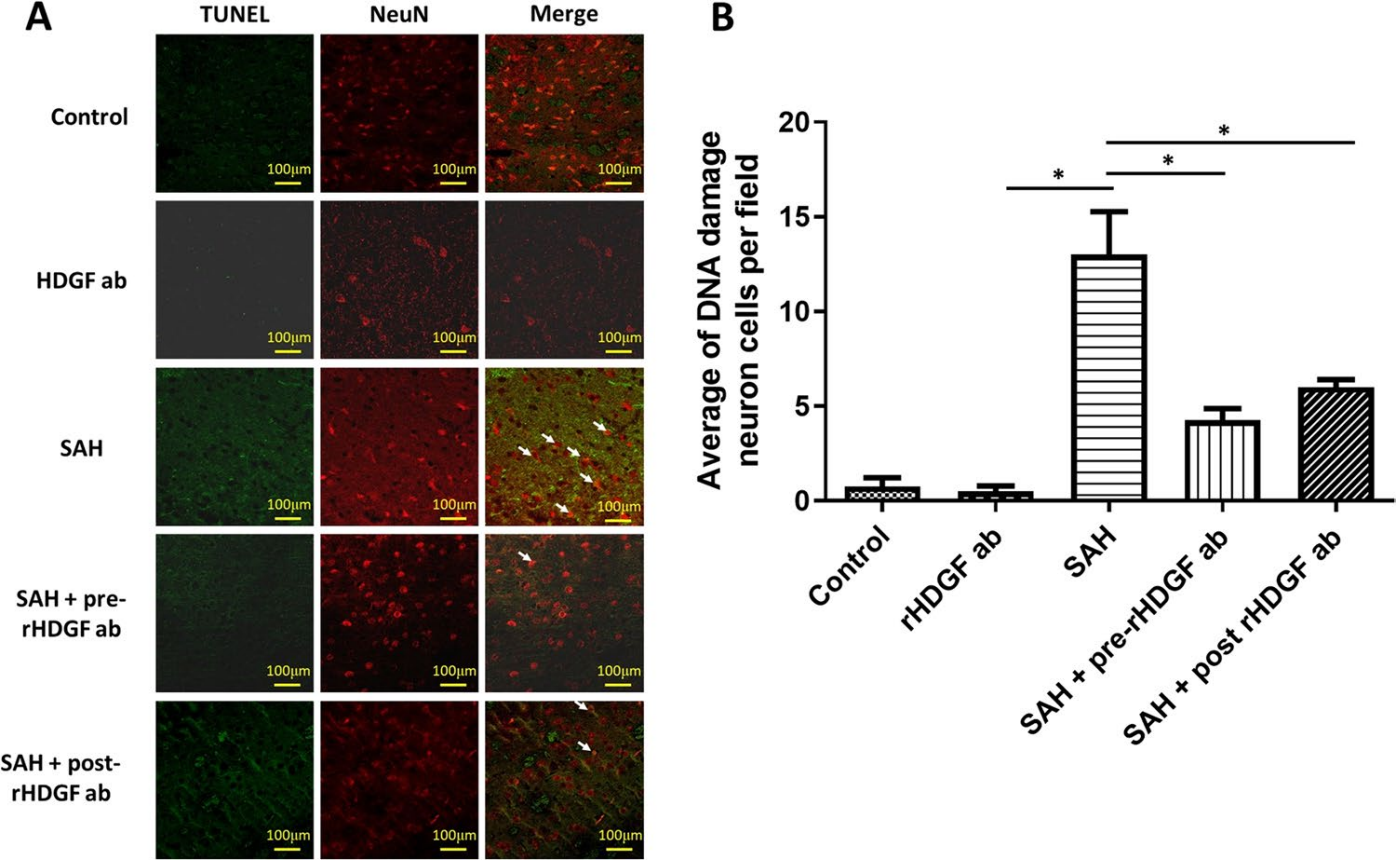
DNA damage of neuron cells in the rat brain as determined by immunofluorescence staining for TUNEL and NeuN. Representative micrographs of immunofluorescence staining are shown for: (**A**) control group, rHDGF ab only group, SAH group, SAH + pre-rHDGF ab, and SAH + post-rHDGF ab group. (**B**) The number of neuron cell with DNA damage from immunofluorescence staining in the images were measured in all groups. All values are mean ± SD (n = 12). *P < 0.05

Apoptosis is a secondary response to DNA damage, and the biological goal is protecting a multicellular organism against a damaged cell [32]. In this study, immunofluorescence staining for TUNEL and neuron cells to detect the DNA damage of neuron cells. Quantitative analysis of the numbers of double immunofluorescence positive staining for TUNEL and NeuN was not significantly different in the control (0.75 ± 0.96) and the rHDGF ab (0.50 ± 0.58) only groups (Fig. 6). SAH induced the DNA damage of neuron cells in the rat brain (Fig. 6A). By contrast, double staining in the brain of the SAH rats was substantially elevated (13.00 ± 4.55), and both pre-treatment with rHDGF ab (4.25 ± 1.26, P < 0.05 compared with the SAH only group) and post-treatment with rHDGF ab (6.00 ± 0.707, P < 0.05 compared with the SAH only group) significantly reduced the DNA damage of neuron cells (Fig. 6B).

## Discussion

It has been reported that HDGF stimulates cell proliferation in fibroblasts, endothelial cells, and hepatoma cells [10] as well as mediates inflammation [33]. Consistent with these findings, our study showed that treatment with rHDGF ab could decreased the proliferation of microglia and astrocytes as well as reduced the levels of pro-inflammatory factors induced by SAH (Figs. 2, 3, and 4).

Inflammation and cytokines may participate in the pathology of blood–brain barrier (BBB) disruption and brain edema, which are characteristic features for both clinical and experimental SAH [34, 35]. A variety of inflammatory cytokines, including IL-1β, IL-6, and TNF-α, are strongly associated with brain injury in the rat [36]. Inhibition of IL-1β has been shown to attenuate early brain injury (EBI) and improve BBB function after SAH [37]. The present study showed that pre-treatment with rHDGF ab decreased the SAH-induced production of TNF-α, IL-1β, and IL-6.

Many studies have shown that HDGF may play a role in apoptosis. For example, silencing the HDGF gene has been demonstrated to prevent TNF-α/cycloheximide-induced apoptosis [38]. In addition, HDGF is found to be essential for TNF α-induced release of pro-apoptotic factors from mitochondria [38]. In contrast, it has been demonstrated that knockdown of HDGF gene induces apoptosis [39, 40] and cell cycle arrest in several human cancers. HDGF knockdown not only induces expression and de-phosphorylation of the pro-apoptotic protein Bad, but also inactivates ERK and Akt, resulting in activation of the intrinsic apoptotic pathway in cancers [40–42]. However, in our study, blocking HDGF attenuated SAH-induced neuron cell apoptosis in the brain (Fig. 6). Similarly, HDGF knockdown triggers the Fas-mediated extrinsic apoptotic pathway in HepG2 cells through the nuclear factor-κB (NF-κB) signaling [43]. NF-κB is a nuclear transcription factor that acts as a key regulator of both inflammatory response and cell death [30, 31]. Our study showed that pre-treatment with rHDGF ab down-regulated inflammatory factors and p-NFκB/NFκB ratio. These data supported the notion that blocking HDGF attenuated apoptosis of neuron cells in the cortex after SAH (Fig. 5D).

Brain-derived neurotrophic factor (BDNF), a molecule that regulates neuronal survival and differentiation, has a critical role in synaptic plasticity [44]. However, BDNF is initially synthesized as a precursor, proBDNF, which is trafficked to the regulated secretory pathway [29]. A previous report showed that mature BDNF can protect neurons from amyloid-beta (Aβ)-induced apoptosis by increasing the expression of bcl-2 [36]. (Irrelevant) Our results showed that the protein levels of proBDNF were significantly elevated in the cortex of SAH rats, and pre-treatment with rHDGF ab decreased proBDNF protein expression following SAH, suggesting that the beneficial effects seen with blocking HDGF may be mediated, at least partially, by the BDNF pathway.

## Conclusion

This is the first study to investigate the relationship between HDGF and SAH. In our results, HDGF induced inflammation and neuron cell apoptosis after SAH. Blocking HDGF reduced SAH-induced vasospasm and attenuated inflammation and cell apoptosis possibly through inhibiting proBDNF and p-NFκB/NFκB pathways.

## Data Availability

Not applicable.
